# Preservation of Auxetic Oscillatory Capacity in Living Connective Tissue: An ECM-Regulated Hypothesis of Mechanical Homeostasis

**DOI:** 10.3390/life16101706

**Published:** 2026-10-12

**Authors:** Brian R. Murer

**Affiliations:** Lazy 8 Healthcare and Consulting, P.C., Bloomington, IN 47401, USA; brm@l8hc.com

**Keywords:** mechanical homeostasis, extracellular matrix, mechanotransduction, biotensegrity, auxeticity, Poisson ratio, auxetic oscillation, deformation capacity, fascia

## Abstract

Living connective tissue preserves function while adapting to changing loads. This hypothesis proposes that preserving access to reversible auxetic deformation can contribute to mechanical homeostasis in selected tissues. Auxetic oscillation (AO) denotes repeated, reversible expression of negative-Poisson behavior; it does not require transitions between Poisson regimes. AO range is defined as the longitudinal-strain interval over which a directional incremental Poisson measure is reliably negative, with loading, unloading, recovery, and cycle stability described separately. The proposed biological contribution is that broader recoverable AO exposure produces cell–matrix inputs that better preserve extracellular-matrix (ECM) adaptability than an appropriately varied non-auxetic comparison. A bounded annulus-fibrosus example predicts less persistent matrix compaction after repeated AO exposure and defined recovery, as a candidate structural indicator of preservation. Equivalent or worse matrix outcomes would count against that advantage. Biotensegrity supplies a candidate architectural rationale for distributed load management, while established tissue mechanics provide the experimental basis. Evidence that auxeticity occurs, that ECM state regulates it, and that preserving it benefits the matrix represents separate levels of support. The contribution is therefore a testable preservation hypothesis, rather than the discovery of a new deformation phenomenon.

## 1. Introduction

Living tissues are mechanically active systems. With every breath, heartbeat, step, change in posture, and muscular contraction, cells and matrices experience changing combinations of tension, compression, pressure, fluid movement, and deformation. Normal tissues preserve function not by remaining mechanically unchanged, but by sensing, adapting, repairing, remodeling, and recovering. This capacity is increasingly described as mechanical homeostasis [1,2].

Mechanical health is often reduced to isolated properties such as stiffness, elasticity, tensile strength, or range of motion. Living connective tissue, however, is nonlinear, anisotropic, viscoelastic, poroelastic, actively remodeled, and dependent on loading history. These established behaviors describe important aspects of tissue mechanics. The present hypothesis asks a different, complementary question: whether preserving access to a negative-Poisson deformation regime, among several available strategies, contributes to the functional operating range of selected tissues [1,3,4].

This article develops a progressive argument. If mechanical homeostasis requires stability during continual change, what architecture can preserve coherence without rigidity? What deformation behaviors may enlarge the repertoire of that architecture? What living material environment regulates those behaviors? Finally, can that relationship be measured and experimentally perturbed?

This article uses auxetic oscillation (AO) for repeated, reversible auxetic deformation: longitudinal and transverse dimensions change in the same direction over identified portions of loading and unloading. The hypothesis concerns preservation of the capacity to access this behavior in selected connective tissues. Section 6 defines that capacity and its measurable range.

The framework is deliberately bounded. It does not claim that all connective tissues are auxetic, equate ECM densification with fibrosis, or assume that a clinical intervention is auxetic merely because it changes motion or perceived tissue quality. These distinctions are necessary if AO is to become an experimentally useful model rather than a metaphor.

### Methodological Approach

This is a narrative hypothesis article, not a systematic review. Topic-driven searches of biomedical and materials-science literature, reference chaining, and updates prompted by peer review informed the synthesis. Sources were selected for relevance to mechanical homeostasis, tissue mechanics, auxeticity, ECM physiology, fluid–structure interaction, and mechanotransduction. No exhaustive search or quantitative evidence grading was performed, and selection bias remains possible. Clinical observations motivated the question but are not evidence of Poisson behavior. Reference checking distinguished bibliographic identity from support for a particular claim; experimental findings, model predictions, and proposed biological extensions are identified separately.

Generative AI assisted conceptual organization, literature-search support, prose revision, development of illustrative testing proposals, and figure preparation. The article presents no AI-generated experimental data. Product information and the scope of assistance are disclosed in the Acknowledgments.

## 2. Mechanical Homeostasis

### 2.1. Mechanical Homeostasis Is Dynamic Regulation

Mechanical homeostasis is commonly described as the maintenance or restoration of a mechanically functional state. In living tissues, however, that state cannot mean a fixed shape, stiffness, tension, or strain. Cells and matrices are continually displaced, loaded, unloaded, hydrated, compressed, and reorganized as the organism breathes, moves, grows, repairs, and adapts. Mechanical homeostasis must therefore preserve function through change rather than prevent change.

Mechanical conditions are biologically instructive as well as structural. Cells adhere to matrices, generate traction, experience deformation and pressure, and alter behavior in response to mechanical cues. Matrix elasticity can influence lineage specification, while matrix cross-linking can amplify integrin-dependent signaling and pathological remodeling [5,6]. Humphrey, Dufresne, and Schwartz accordingly described connective tissues at steady state as dynamic systems in which resident cells continually sense their environment and modify ECM synthesis, degradation, organization, and prestress [1].

Tschumperlin and colleagues extended this homeostatic framing to injury and fibrosis: an injury perturbs the mechanical environment, whereas unresolved mechanosensitive feedback can stabilize pathological matrix deposition [2]. Homeostasis is therefore not synonymous with return to an identical prior state. Successful regulation may involve restoration, compensation, or remodeling, provided that the resulting mechanical conditions remain compatible with tissue function.

### 2.2. The Regulated Variable May Be an Operating Range

The mechanically regulated quantity need not be one universal scalar value [3,4]. A tissue can remain functional across changing combinations of prestress, stiffness, compliance, strain distribution, fluid movement, energy storage, dissipation, and recovery. The relevant homeostatic target may therefore be an operating range: a bounded repertoire of mechanical states that the tissue can enter, tolerate, and leave without losing integrity or adaptive capacity.

We use mechanical adaptability for the capacity to access and recover among such functional states under changing loads. This capacity does not require maximal compliance or minimal stiffness. Transmission may require stiffness; shape change requires sufficient compliance; recovery depends on loading history, fluid redistribution, fibrillar recruitment, cellular prestress, and time. A mechanically adaptable tissue must coordinate these properties rather than maximize any one of them.

### 2.3. Nested Regulation Across Scales and Timescales

Mechanical homeostasis is unlikely to be governed by a single controller. Molecular interactions, hydrated ground substance, fibrillar architecture, cellular mechanosensing, muscle activity, neural regulation, vascular exchange, immune signaling, metabolism, and ECM turnover operate concurrently. Their characteristic timescales differ: fluid and molecular redistribution may begin during loading, cellular signaling may alter activity over seconds to hours, and synthesis, degradation, and architectural remodeling may continue over days to months. These processes are nested because changes at one level modify the conditions experienced at others.

Lima and Blazevich provide a useful example of this nested organization. Their analysis separates molecular, ECM, tissue, neural, perceptual, and behavioral contributors to passive stiffness and voluntarily expressed range of motion [7]. Although they do not describe these relationships as mechanical homeostasis, their synthesis demonstrates why neither range of motion nor bulk stiffness can serve as a complete proxy for the state of the system. Their more specific implications for interpreting treatment and performance outcomes are considered in Section 8.6.

### 2.4. Reciprocal Feedback, Adaptation, and Dysregulation

The nested relationships are reciprocal rather than arranged as a one-way hierarchy. Present tissue state influences how load is distributed; deformation alters cellular and sensory input; neural and muscular responses change subsequent loading and prestress; and repeated exposure can modify ECM composition and architecture. The altered matrix then conditions the deformation available during the next loading cycle. Mechanical homeostasis emerges from this continuing feedback rather than from command by any single component.

The same reciprocity permits dysregulation. A compensatory response that is useful in the short term may become restrictive if it persists, while structural remodeling can preserve function in one context yet narrow the range of states available in another. The important distinction is therefore not simply normal versus abnormal stiffness, but whether the system retains a task-appropriate capacity to reconfigure and recover.

### 2.5. The Architectural Requirement

This formulation creates an architectural problem. A living system must preserve coherence while its geometry, internal loading, and boundary conditions change. Established anisotropic fiber-reinforced, viscoelastic, and poroelastic mechanics provide the primary material basis for analyzing these responses. Biotensegrity offers an additional interpretative framework for considering how prestress and distributed relationships may support reconfiguration across connected structures.

## 3. Biotensegrity: Architecture for Mechanical Homeostasis

### 3.1. Biotensegrity as a Candidate Architectural Interpretation

Classical tensegrity describes prestressed structures whose stability arises from an integrated relationship between tensile and compression-resisting elements. Levin extended this reasoning to living systems through the concept of biotensegrity: distributed force vectors, hierarchical organization, prestress, and continual reconfiguration [8,9,10,11]. Here, biotensegrity is used as a candidate architectural interpretation rather than as a substitute for established tissue mechanics. It asks how coherence might be preserved while loads and configurations change.

### 3.2. Defining Architectural Characteristics of Tensegrity Systems

Prestress supplies an initial mechanical state before a new external load is applied. Connected elements are therefore already engaged, and a local perturbation can be transmitted, redirected, shared, or contained through more than one pathway. The response depends on geometry, the distribution of prestress, and the boundaries acting on the system [8,9,10,11,12].

Geometry and hierarchy make those pathways adaptable. Changes in angle, spacing, orientation, or constraint alter which elements are recruited and how force is resolved; apparent stiffness may therefore change through reconfiguration even when constituent material properties do not. A component may also contribute differently across position, load, scale, and time rather than remain exclusively tensile or compression-resisting [8,9,10,11,12].

At the cellular scale, experimentally developed tensegrity models describe mechanical continuity from ECM adhesions through the cytoskeleton to the nucleus [13,14]. This provides a biological precedent for the narrower proposition used here: prestress, connectivity, and configuration can influence both force transmission and mechanosensitive signaling.

### 3.3. Relevance to Mechanical Homeostasis and AO

Biotensegrity provides an architectural rationale for investigating auxetic capacity. Levin’s application of prestress, connectivity, and distributed force paths to biology motivates the possibility that coordinated dimensional change can preserve engagement as a system reconfigures [8,9,10,11]. The link between tensegrity and auxeticity also has a specific materials-science precedent: Oster and colleagues demonstrated auxetic behavior in a designed reentrant tensegrity structure [15]. That result concerns a particular engineered geometry; it does not establish that every tensegrity or connective tissue is auxetic.

Applied to tissue, the proposed connection is that reversible auxetic reconfiguration could help differently oriented elements remain engaged while sharing load. Cellular tensegrity research supplies a biological precedent for configuration-dependent force transmission and mechanosensing [13,14]. The AO-preservation hypothesis extends this reasoning to a measurable tissue capacity whose effects on load distribution and matrix function require testing. Auxeticity is neither diagnostic of biotensegrity nor dependent on acceptance of the organism-level model.

## 4. Auxetics: Characteristics, Advantages, and Biological Evidence

### 4.1. Definition and Measurement Boundaries

Auxeticity describes a specific relationship between longitudinal and transverse deformation. Under an appropriate material test, a structure demonstrates negative-Poisson behavior when longitudinal extension is accompanied by transverse expansion. Figure 1 illustrates transverse narrowing during longitudinal extension in a positive-Poisson response and transverse widening during longitudinal extension in a negative-Poisson response. Reversing the direction of movement does not necessarily end that relationship: if longitudinal shortening during unloading is accompanied by transverse narrowing, the incremental strain relationship may remain negative-Poisson. Repeated loading and unloading within an auxetic regime must therefore be distinguished from an actual transition between negative- and non-negative-Poisson behavior. AO concerns repeated, reversible access to the auxetic relationship; it does not require every cycle to cross between Poisson regimes.

A biological tissue need not have one fixed Poisson response. Depending on direction, mechanical state, and boundary conditions, the same tissue may display negative, positive, or near-zero behavior. The present hypothesis treats this flexibility as part of the tissue’s deformation repertoire. Positive-Poisson and other non-auxetic responses may be useful under some conditions, while preserved auxetic capacity provides an additional strategy when its mechanical requirements are met. Auxeticity should therefore not be assigned as a permanent identity to an entire tissue.

**Figure 1:**
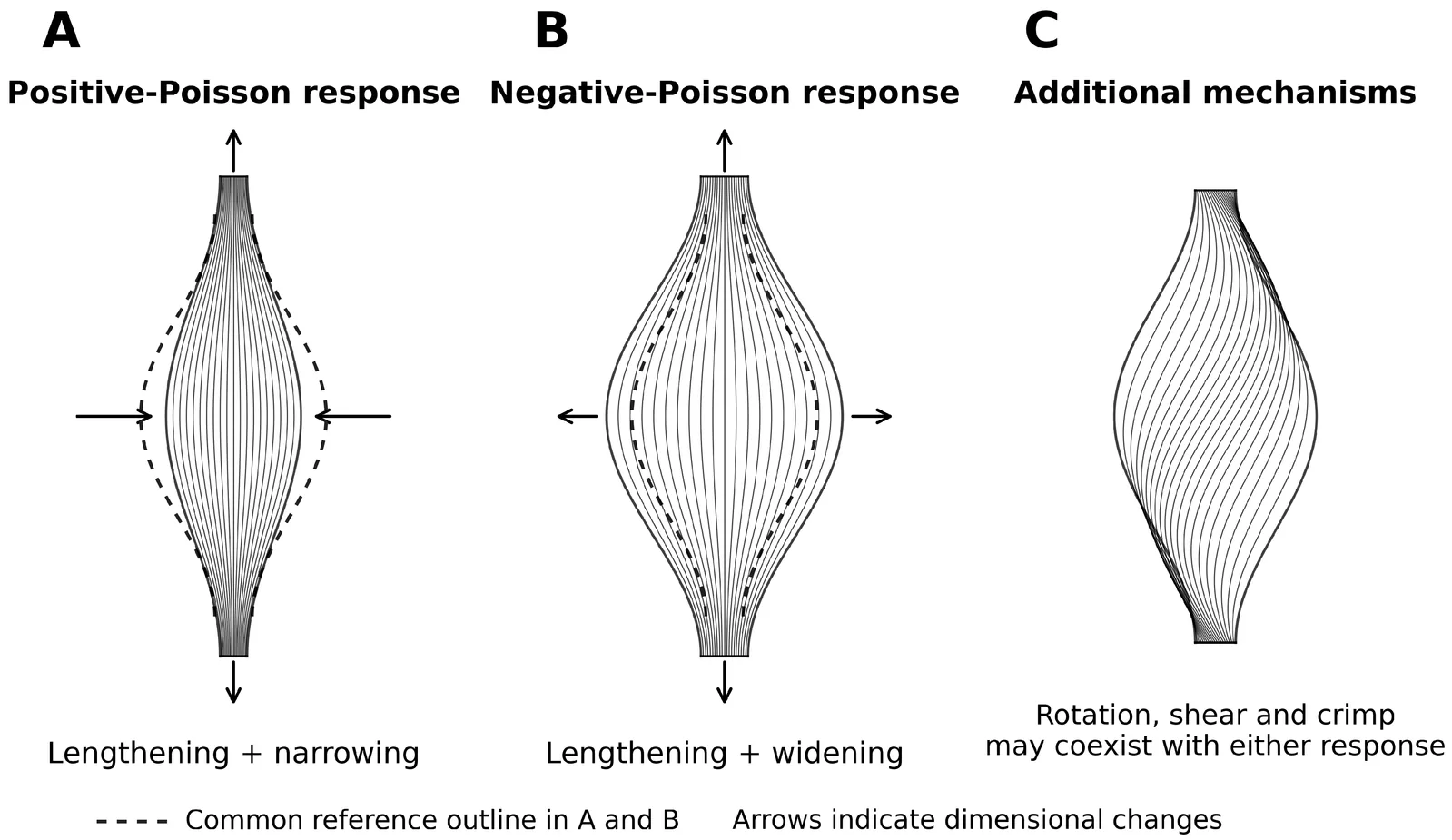
Alternative dimensional responses and additional deformation mechanisms. Panels (**A**,**B**) illustrate the same longitudinal extension from a common dashed reference outline. Extension accompanies transverse narrowing in (**A**) and widening in (**B**). Arrows indicate dimensional changes, not independently applied transverse forces. Panel (**C**) illustrates rotation and nonuniform reconfiguration, which may coexist with either Poisson response. Retaining access to the response in (**B**) is the capacity investigated here; the drawing does not establish its biological advantage.

A final distinction is necessary between a material Poisson response and apparent strain coupling in vivo. Poisson behavior is most defensibly identified when longitudinal and transverse strains are measured within a specified tissue, reference configuration, and loading condition. Under anatomical constraint or multiaxial loading, directional apparent strain coupling may be the more accurate description. Local tissue strain must also be distinguished from translation, rotation, movement of surrounding structures, or deformation introduced by the measuring instrument. Claims about volume, pore space, or fluid movement should, where feasible, include both transverse axes rather than infer three-dimensional behavior from a single plane [16].

### 4.2. Architecture-Dependent Advantages in Materials Science

In engineered materials, auxetic behavior arises primarily from internal architecture rather than the chemical composition of the constituent material alone. Re-entrant geometries, rotating units, chiral structures, folding patterns, and hierarchical lattices can convert longitudinal loading into coordinated transverse deformation [17,18,19]. This response can produce combinations of properties that are difficult to obtain through a simple increase in stiffness.

Reported advantages include increased indentation resistance, improved distribution of localized loads, enhanced fracture toughness in selected configurations, synclastic curvature, adjustable porosity and permeability, and useful forms of impact attenuation, damping, and energy absorption [18,19,20,21,22]. Experimental precedents include strain-dependent indentation resilience in auxetic microporous polyethylene [20], mechanically tunable filtration in an auxetic architecture [21], and enhanced fracture toughness in re-entrant metallic foams under defined processing conditions [22]. None is guaranteed by a negative Poisson ratio alone. Their expression depends on architecture, density, constituent material, loading direction, strain range, deformation rate, boundary conditions, and whether deformation is elastic, viscoelastic, or plastic.

The common mechanical feature is coupling of longitudinal and transverse deformation. During longitudinal tension, an auxetic architecture recruits material transversely rather than narrowing away from the loading direction. During compression, it may contract along multiple axes rather than spreading conventionally. This coupling changes how material is recruited around a load, how internal spaces change, how curved surfaces are accommodated, and how stress and strain are distributed. Materials science therefore identifies mechanical possibilities that may arise when a hierarchical biological structure can reversibly couple longitudinal extension to transverse expansion.

### 4.3. Potential Advantages in Living Fascia and Connective Tissue

#### 4.3.1. Load Distribution and Recoverable Recruitment

Auxetic reconfiguration may recruit material transversely while a tissue lengthens, providing an additional route for accommodating load. In selected engineered architectures, this recruitment improves resistance to indentation or changes the stress field around a defect [20,22]. A corresponding tissue-level advantage would be a reduction in focal strain or pressure while preserving recoverable deformation. It cannot be inferred from widening alone: force transmission, tissue integrity, and recovery must be assessed alongside the strain response.

Within a biotensegrity interpretation, the relevant possibility is continued engagement of connected elements through changing geometry. That may assist distributed load management without requiring generalized rigidity. Energy storage, recoverable return, dissipation, and damage remain distinct outcomes; a negative Poisson response does not maximize all of them. The biological proposal is task-appropriate recruitment and recovery, rather than universal mechanical superiority.

#### 4.3.2. Surface Geometry, Spaces, and Transport

Auxetic sheets and porous architectures can exhibit useful curvature and strain-dependent pore changes [17,21]. Comparable behavior in fascia could help an envelope accommodate compound shape change or alter spaces between fibers and tissue layers. Whether this preserves space around embedded vessels or nerves, improves transport, or reduces local failure has not been established as a consequence of auxetic deformation in native fascia.

Ground-substance composition, fixed charge, hydration, and pore geometry already influence swelling and fluid movement in tissue-specific ways [23,24,25,26]. Auxetic expansion could modify those conditions, with recovery restoring some of the previous geometry. Tissue volume and transport cannot, however, be inferred from one transverse dimension. These proposed advantages require three-dimensional deformation or independent fluid-sensitive measurements.

#### 4.3.3. The Cell–Matrix Environment

Different combinations of longitudinal strain, transverse strain, fiber recruitment, pressure, and fluid movement expose cells to different mechanical inputs. Auxetic scaffold research provides a reason to investigate these differences, not proof of the same effects in native fascia [17]. The distinctive proposal is that preserving a recoverable AO range helps maintain future matrix adaptability. Section 8 separates a response to different inputs from a comparative biological benefit and from the usefulness of AO measures as predictors.

### 4.4. Biological Evidence and Counterexamples

Direct evidence for negative or condition-dependent Poisson behavior in biological tissues remains selective, with much of the existing work focused on tendon. Gatt and colleagues reported negative Poisson ratios in human and animal tendons under defined conditions [27]. Carniel and colleagues characterized transverse deformation during tendon tensile testing and emphasized the importance of specimen configuration, measurement method, and constitutive interpretation [28]. Modeling further suggests that fiber orientation and crimp can influence effective Poisson behavior [29]. At a higher level of tendon organization, interfascicular-matrix sliding permits adjacent fascicles to move relative to one another and contributes to transverse deformation, viscoelastic energy dissipation, and the distribution of strain before failure [30]. These findings suggest that tendon’s effective Poisson response may emerge from coordinated behavior across several structural levels rather than from any single constituent or mechanism. Evidence also extends beyond tendon. Annulus-fibrosus studies combine experimental strain measurements with constitutive interpretation of directional, osmolarity-dependent, and time-dependent responses [31,32]. These studies already encompass important features now grouped under AO: cyclic or time-varying transverse behavior is not claimed as a newly discovered phenomenon.

Direction-dependent behavior has also been measured in skin. Dwivedi and colleagues found lateral contraction when skin was stretched parallel to skin-tension lines and lateral expansion in perpendicular ex vivo specimens, relating the response to collagen-fiber rotation and fluid movement; the in vivo findings were more constrained and did not reproduce the same auxetic response [33]. This contrast is particularly relevant to AO because it demonstrates that tissue architecture may permit negative-Poisson behavior while anatomical constraint determines whether it is expressed.

Shokrani and colleagues reported auxetic circumferential responses in colorectal tissue and quantified deformation transmitted to embedded sensory nerve-fiber networks [34]. This study extends the biological evidence beyond load-bearing tendon and provides a direct example in which transverse tissue behavior changes the mechanical environment of embedded neural structures. It supports the plausibility of a deformation-to-mechanotransduction bridge, although it does not establish the proposed ECM feedback or the superiority of an auxetic regime.

Related observations must still be weighted separately. Price and colleagues measured position-dependent plantar-fascia thickness and shear-wave behavior across rearfoot positions [35]. Because the study did not directly measure the longitudinal and transverse tissue strains required to establish a Poisson ratio, it remains hypothesis-generating rather than direct evidence of auxeticity. Counterexamples are equally important: improved arterial-wall measurements rejected an auxetic interpretation in that preparation [36]. Collectively, the literature supports tissue-, direction-, and constraint-dependent behavior; it does not show that all fascia is auxetic or that negative-Poisson behavior is always preferable.

### 4.5. From Architectural Possibility to ECM Regulation

The architectural link developed in Section 3.3 makes auxetic capacity worth measuring, but a local response cannot establish regional or whole-body auxeticity. Neighboring structures may widen, narrow, rotate, or redistribute fluid during the same task. Coordinated behavior need not be uniform.

Table 1 separates direct observations, mechanical models, related tissue findings, and proposed benefits. The biological question is therefore what permits a tissue to access a recoverable auxetic regime, what restricts that access, and whether preserving it alters the future matrix state. These questions direct attention to the ECM.

## 5. The Extracellular Matrix as an Adaptive Material Environment

### 5.1. A Dynamic Material and Regulatory Environment

The extracellular matrix is a dynamic material and regulatory environment. It bears and redistributes load, maintains hydrated spaces, permits molecular transport, presents ligands to cells, and records previous mechanical conditions. Cells modify it through synthesis, degradation, alignment, cross-linking, and contractile prestress [1,13,38]. Guimberteau and colleagues’ in vivo observations of the microvacuolar sliding system provide a complementary morphological account of living connective tissue maintaining continuity while reconfiguring during movement [37]. That work supports a dynamic, hydrated, reconfigurable substrate; it does not establish auxeticity or AO.

This reciprocal relationship is central to mechanical homeostasis. The state of the ECM influences which deformations a tissue can access; those deformations determine the mechanical and transport environment experienced by resident cells; and cellular responses subsequently alter the matrix that will govern future loading. Frantz, Stewart, and Weaver reviewed the broader biological importance of this reciprocity, describing how changes in matrix composition, organization, and mechanics can redirect cellular behavior and participate in disease progression [39]. The question developed here is narrower: can ECM state also regulate a tissue’s capacity to enter and recover from auxetic deformation?

### 5.2. Fibrous Architecture Defines the Available Geometry

In materials science, auxetic behavior commonly emerges from architecture rather than chemical composition alone. The biological counterpart is not a single re-entrant unit but a hierarchy of collagenous, elastic, and non-collagenous relationships whose geometry changes under load. Collagen orientation and crimp determine the directions and sequence in which fibers are recruited. Lamellar arrangement, fiber rotation, cross-linking, and interfibrillar or interfascicular mobility determine whether recruitment can be accompanied by sliding, separation, or transverse expansion. These variables do not merely set tissue strength; together they define the geometric pathways available during deformation.

Evidence from annular and tendinous tissues supports this architectural principle: fiber orientation and crimp can alter effective Poisson behavior, while interfascicular mobility contributes to transverse deformation and viscoelastic recovery [29,30]. Architecture defines the available pathways; the surrounding material environment helps determine whether they can be accessed.

### 5.3. Ground Substance, Hydration, and Osmotic Regulation

Fibrous architecture operates within hydrated ground substance. Glycosaminoglycans, proteoglycans, hyaluronan, ions, glycoproteins, and water can contribute to fixed-charge behavior, osmotic swelling pressure, lubrication, permeability, and resistance to compression, although their relative contributions differ substantially among tissues [23,24,25,26]. Cartilage provides the best-established example of fixed-charge and osmotic swelling mechanics [26]; applying the same quantitative behavior to fascia would be an inference, not an established equivalence. Ground substance should therefore be understood not as filler between fibers, but as part of the tissue-specific mechanical conditions under which fibers recruit and recover.

This provides a plausible connection between ECM state and auxetic capacity. A collagen network with sufficient interfibrillar mobility and hydrated spacing may have greater freedom to rotate, unfold, separate, and change transverse dimensions during longitudinal loading. Altered hydration, molecular crowding, or hydraulic resistance could restrict the same pathway even if the gross collagen arrangement remained recognizable. Annulus-fibrosus experiments showing osmotic and rate dependence of transverse behavior offer a direct precedent for this state dependence [31]. They do not prove a general pathway across fascia, but they demonstrate that Poisson behavior can be altered by the physicochemical environment in which fibrous architecture is loaded.

### 5.4. Prestress and the Initial Mechanical State

A tissue’s response is determined not only by its components but also by the configuration from which loading begins. Cellular contractility, muscle activity, residual strain, intratissue pressure, adjacent anatomical constraints, and previous loading establish prestress and boundary conditions before an external test or movement begins. Fiber populations already recruited at baseline cannot respond in the same way as fibers that remain crimped or mechanically disengaged. Thus, identical external loads need not produce identical deformation trajectories.

This initial-state dependence helps explain why positive-, near-zero-, and negative-Poisson behavior may occur within the same tissue under different conditions. Auxeticity need not be a permanent label; it may be a regime entered only when architecture, hydration, prestress, and boundary conditions permit a particular reconfiguration. The same principle also separates tissue state from tissue composition. A rapid change in prestress or fluid distribution may alter mechanical behavior without requiring immediate synthesis or removal of collagen.

### 5.5. Mechanical State and Structural Remodeling Operate Across Timescales

The ECM can change mechanically before it changes compositionally. During and immediately after loading, fiber recruitment, crimp reduction, relative sliding, pore deformation, pressure gradients, fluid redistribution, and mechanosensitive ion flux may change the tissue’s operating state. Over minutes to hours, viscoelastic recovery, cytoskeletal reorganization, focal-adhesion dynamics, and altered signaling can modify how subsequent loads are received. Over days to months, synthesis, degradation, cross-linking, fiber reorganization, and persistent changes in cellular phenotype can remodel the matrix itself.

These timescales should not be treated as competing explanations. A reversible short-term state change may be physiologically useful precisely because it alters the signals that guide longer-term adaptation. Conversely, repeated loading without adequate recovery may stabilize a transiently restricted state through cellular and matrix remodeling. For AO, this distinction is essential: an immediate change in deformation does not establish durable architectural remodeling, but neither should the absence of immediate remodeling be interpreted as absence of a tissue-level response.

### 5.6. Mechanotransduction Links Deformation to Future ECM State

Mechanical deformation first changes the physicochemical environment in which cells and fibers are embedded. Compression, tension, shear, and multiaxial reconfiguration can alter pore dimensions, hydrostatic and osmotic pressures, fluid flow, ion distribution, molecular crowding, and the transport of metabolites, cytokines, and matrix fragments. Glycosaminoglycans, proteoglycans, hyaluronan, fixed charge, and bound water therefore participate in the mechanical signal rather than merely surrounding it [23,24,25,26]. The same gross longitudinal displacement may produce a different ground-substance environment when transverse narrowing, expansion, or shear differs.

At the cell surface, integrins and focal adhesions couple extracellular ligands to the cytoskeleton, while mechanosensitive ion channels and membrane-associated complexes respond to deformation, tension, pressure, and fluid shear. These inputs can change calcium signaling, focal-adhesion assembly, cytoskeletal organization, cellular contractility, and the forces that cells return to the matrix [1,13,14,38]. Mechanotransduction is therefore reciprocal: matrix state conditions cellular loading, and cellular traction immediately modifies the mechanical state of the surrounding matrix.

Mechanical information can also reach the nucleus through cytoskeletal continuity and signaling pathways. Changes in nuclear deformation and transcriptional regulators can alter inflammatory activity, growth-factor signaling, matrix synthesis, matrix-metalloproteinase activity, cross-linking, and myofibroblast differentiation [1,2,5,6,13,14,38,40,41]. These responses unfold over different timescales. Ion movement, fluid redistribution, and cytoskeletal tension may change during or soon after loading, whereas altered gene expression, protein synthesis, and durable architectural remodeling develop more slowly.

The relevant stimulus is unlikely to be strain magnitude alone. Direction, rate, pressure, fluid movement, repetition, and recovery shape the physicochemical environment. Trajectories with similar longitudinal strain can therefore elicit different cellular inputs. The AO-preservation hypothesis asks whether the resulting exposure pattern helps maintain matrix adaptability, and whether AO descriptors summarize biologically useful features of that pattern.

### 5.7. Adaptation, Densification, and Fibrosis

An adaptable matrix is not defined by low stiffness or continuous expansion. It must provide sufficient resistance for force transmission while permitting the task-appropriate movement of fibers, fluid, cells, and tissue interfaces. Dysfunction may therefore begin as a narrowing of the accessible operating range rather than as an obvious increase in bulk stiffness. Changes in hydration, packing, molecular interaction, or cellular tension may increase resistance to physiological deformation or relative tissue movement without yet constituting fibrosis.

Here, densification is used only as a descriptive, operationally testable concept: greater matrix packing or reduced mobile/interstitial space accompanied by increased resistance to relative tissue or fluid movement. It should be identified with independent structural or compositional evidence appropriate to the tissue, not inferred from palpation, stiffness, pain, or reduced motion alone. A transient hydration change must be separated from persistent architectural remodeling. Fibrosis is a distinct pathological program involving altered matrix deposition, organization, stiffness, and sustained profibrotic signaling [2,40,41]. The terms may overlap in some conditions but are not interchangeable.

### 5.8. The ECM-to-Auxetic-Capacity Proposition

ECM state may regulate auxetic capacity by changing geometry, hydration, mobility, prestress, or recovery. Association alone is insufficient because these features can change together. A regulatory claim requires a controlled perturbation of a defined ECM feature, independent confirmation that it changed, and control or measurement of accompanying mechanical variables. Acute reversible state changes must be distinguished from slower remodeling.

The reciprocal extension is that repeated deformation also changes the signals available to cells and thereby may alter future ECM state. This is the preservation hypothesis developed next: the matrix permits a deformation strategy, and repeated access to that strategy may help sustain the matrix conditions needed for future reconfiguration.

## 6. Auxetic Oscillation: An Integrative Mechanobiological Hypothesis

### 6.1. AO Behavior, Capacity, and Range

Auxetic oscillation (AO) denotes repeated, reversible expression of a negative-Poisson response over identified portions of tissue loading and unloading. Longitudinal and transverse strain increments have the same sign in these portions: lengthening accompanies widening, or shortening accompanies narrowing. Reversal of movement does not itself indicate departure from an auxetic regime. AO names a dynamic behavior already compatible with established tissue mechanics; the hypothesis concerns the value of preserving access to it.

For the illustrative material test, the primary descriptor is the directional incremental Poisson measure: the negative of the change in local transverse strain divided by the change in local longitudinal strain over a resolved increment. Engineering strains are referenced to the same equilibrated, preloaded configuration, with axes fixed in the tissue. Loading and unloading are evaluated separately. Unresolved increments and turning points at which longitudinal change approaches zero are excluded. This specifies a comparison convention rather than a constitutive model; studies using another finite-strain measure must identify it and cannot assume numerical equivalence [16]. Under imposed multiaxial anatomical constraints, the corresponding result is described as apparent directional strain coupling.

AO range is the width of the longitudinal-strain interval over which the chosen incremental measure is reliably negative. Entry and exit are the first and last qualifying increments on each branch, using a prespecified resolution criterion. Disconnected intervals are reported separately; gaps are not counted as auxetic range. Transverse magnitude, duration of exposure, and access under different rates or boundaries are separate descriptors, not alternative meanings of range.

AO capacity is the reproducible ability to access that range and recover under stated conditions. Recovery is assessed by residual longitudinal and transverse strain relative to the precycle reference at a specified elapsed time, and by the time required to return within a tolerance set before testing. Cycle drift is the change in range or transverse response between specified repeated cycles. Loading–unloading separation in the strain–strain path describes kinematic hysteresis; energy dissipation requires force or stress measurements and is not inferred from that separation alone.

A broader AO exposure therefore spans more of a common, nondamaging longitudinal-strain window while meeting the same recovery criterion. Limited exposure occupies a smaller qualifying span; absent exposure has no resolvable qualifying interval under those conditions. Threshold, response magnitude, hysteresis, recovery time and cycle drift complete the exposure profile. Broader access across different loading conditions is reported separately. These definitions avoid treating the most negative response or the largest possible deformation as the physiological optimum.

Figure 2 illustrates reversible deformation within an auxetic regime and how different mechanical constraints can permit positive- or negative-Poisson responses.

### 6.2. AO and the Adaptable Extracellular Matrix

AO and ECM state are proposed to have a reciprocal relationship. The matrix determines whether auxetic reconfiguration is available and recoverable; the resulting strain and fluid environment shape the inputs received by cells. Repeated access to an appropriate AO range may support the hydration, mobility, and organization needed for subsequent deformation. This preservation advantage, rather than auxeticity alone, is the biological claim.

Persistent restriction could expose cells to a different pattern of loading and recovery, favor a less reconfigurable matrix, and further reduce AO access. Positive-Poisson behavior, rotation, shear, and fiber crimp remain useful and often concurrent mechanisms. The proposed risk concerns chronic loss of an otherwise available capacity, not any isolated non-auxetic event.

**Figure 2:**
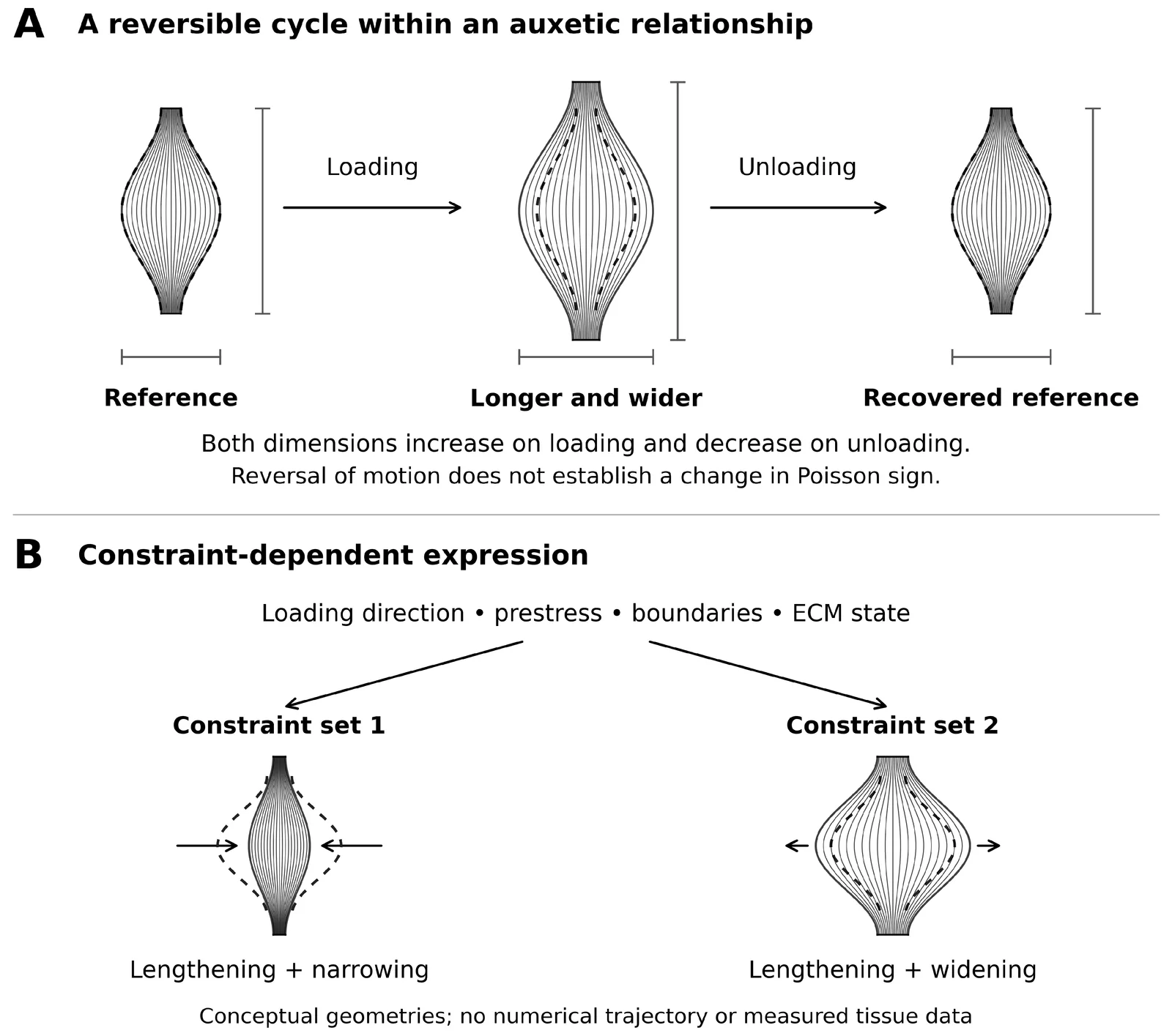
Reversible auxetic deformation and constraint-dependent expression. (**A**) Longitudinal and transverse guides identify dimensional changes from a common reference. Both dimensions increase during loading and decrease during unloading; motion reverses without requiring a change in Poisson sign. (**B**) Different specified constraints can permit narrowing or widening during longitudinal extension. These alternatives do not imply that transitions must be abrupt or that Poisson behavior cannot vary continuously with changing conditions. Arrows indicate loading and unloading or the direction of the depicted dimensional changes; branching arrows in panel (**B**) indicate alternative constraint-dependent responses. Dashed lines provide reference guides for comparing longitudinal and transverse dimensions. Measurement key: AO range is the qualifying longitudinal-strain span, reported separately for loading and unloading; recovery is residual deformation at a stated time; drift is change across repeated cycles.

### 6.3. AO and Distributed Load Management

Within a prestressed, connected architecture, longitudinal extension with transverse widening may recruit a broader field of structural relationships than extension with narrowing. This supplies the proposed link between auxetic capacity and biotensegrity’s distributed load management. Whether that recruitment actually reduces local concentration or preserves tissue strength requires independent force, strain-distribution, and integrity measurements; widening by itself establishes none of these outcomes.

### 6.4. The Comparative Preservation Hypothesis

The central prediction is that repeated exposure across a broader recoverable AO range will better preserve a specified matrix property than both a restricted low-variation exposure and an appropriately varied non-auxetic exposure. The latter comparison is essential: otherwise an apparent advantage could result from greater loading variety rather than auxetic access.

Matrix preservation must be assessed independently of AO capacity. Section 8.5 proposes less persistent matrix compaction after repeated loading and common recovery as a candidate structural indicator of preservation, with tissue integrity and viability maintained. Its relationship to functional adaptability requires independent assessment. General ECM–cell reciprocity supports investigating this possibility [39], but does not establish the proposed advantage.

## 7. Proposed Sequence of AO Failure and Loss of Mechanical Homeostasis

The proposed failure sequence is a feedback process. Injury, altered loading, immobilization, inflammation, aging, or changes in hydration and prestress may shift the matrix conditions that permit recoverable deformation. In a tissue with demonstrated AO access, this could narrow the qualifying strain interval, change its magnitude, or slow recovery before a large change in bulk stiffness occurs.

The tissue may then rely more often on other deformation strategies. Such compensation can be useful. The hypothesis is that persistent loss of the auxetic option changes cell–matrix inputs in ways that promote restrictive adaptation, further reducing future access. Matrix packing, fluid redistribution, fiber recruitment and cellular remodeling could participate on different timescales. A transient water shift is not equivalent to lasting densification, and fibrosis is neither required nor assumed.

Figure 3 separates established general reciprocity among matrix, mechanics, and cells from the proposed AO-specific preservation and restriction loops. Both loops are hypotheses. Their evaluation requires independently measured matrix outcomes and temporal evidence, rather than interpreting an immediate change in tissue shape as proof of remodeling.

## 8. Experimental and Translational Implications

The research sequence is to establish a reproducible tissue response, test whether a defined ECM change regulates it, and determine whether preserving it changes an independent biological or matrix outcome. Table 2 distinguishes these evidentiary levels. The examples below are prospective tests, not completed experiments or validated clinical protocols.

### 8.1. Measurement and Exposure Classification

Concurrent local longitudinal and transverse strain measurements are required, using the reference state, axes, branch-specific incremental convention, and recovery criteria defined in Section 6.1. The negative Poisson measure corresponds to same-sign strain increments, not a negative transverse-versus-longitudinal slope. Rotation and translation must be separated from local strain. Both transverse axes are needed when interpreting volume change; fluid movement requires independent measurement rather than inference from visible widening alone.

Report the qualifying strain span, sign and magnitude of coupling, entry and exit, access conditions, loading–unloading separation, recovery time, and drift across identified cycles. Record the uncertainty needed to classify a response as present, limited, or absent. Reference configuration, boundaries, hydration, temperature, preload, strain amplitude, loading rate, cycle number, and rest interval must accompany the results. The definitions do not require a particular imaging platform. Elastography, range of motion, palpation, and symptoms cannot substitute for the necessary strain measurements.

### 8.2. ECM Association and Regulation

The regulatory criteria in Section 5.8 require a controlled, independently verified ECM change with accompanying mechanical variables addressed. The experiment must distinguish acute hydration or osmotic effects from persistent changes in matrix organization or composition.

Annular studies already connect osmotic conditions and time dependence with transverse deformation [31,32]. A bounded perturbation study could compare paired preparations from one region before and after a controlled osmotic change, independently confirming water-content change and including a time-matched bath control and restoration to the initial bath. Reversal or rescue strengthens interpretation, but bath composition can act directly on cells as well as through mechanics. This acute experiment therefore tests ECM regulation of the mechanical response; it must not, by itself, be presented as a test of AO’s biological benefit.

### 8.3. Biological Response, Mediation, and Predictive Value

Auxetic and non-auxetic trajectories differ in their transverse inputs. A signaling difference therefore shows that cells respond to different mechanical environments, not necessarily an AO-specific feedback mechanism. The biological claim is stronger when repeated exposure precedes the predicted independent matrix outcome and the comparison excludes plausible alternative causes.

AO descriptors can also be tested for prespecified explanatory or predictive value beyond a practical baseline containing longitudinal-strain exposure, stiffness, pressure, and fluid-dependent measures. The baseline, outcome, and validation approach should be specified before analysis. Because AO descriptors are derived from strain histories, they are candidate summaries of those histories, not an independent force or information source beyond an otherwise complete description.

Fluid movement and pressure may mediate the proposed effect. An effect explained through those pathways would narrow its mechanism rather than automatically refute AO preservation. Conversely, an outcome caused by an uncontrolled boundary or bath change cannot be attributed to AO. Evidence for a biological effect, for a beneficial effect, and for added predictive usefulness should therefore be reported separately.

### 8.4. Comparative Loading Conditions

The principal comparison includes broader recoverable AO exposure, a varied non-auxetic exposure, and a restricted low-variation exposure. Broad and limited AO are classified by the qualifying strain span within a common longitudinal-strain window, with the remaining profile reported separately. Restricted low variation is a different classification: it describes reduced variation in direction or trajectory and must not be treated as synonymous with a narrow AO span.

Mechanical dose must account for strain amplitude, rate, cycle number, preload, total exposure, and recovery intervals. Variation in the non-auxetic condition should cover the same planned range of directions and rates where mechanically feasible, rather than being represented by a single repetitive path. The intended transverse difference cannot simultaneously be matched away. Record it, along with pressure, fluid movement and loading work; recognize that matching strain and matching stress may require different comparisons.

Changes to architecture or constraints used to obtain the different responses must also be characterized. If groups differ systematically in substrate chemistry, baseline stiffness, cellular viability or other direct biological inputs, the comparison tests a combined intervention and cannot isolate the benefit of auxetic access. These limitations define the strength of the inference rather than justify changing the hypothesis after observing the result.

### 8.5. A Primary Annulus-Fibrosus Prediction

A bounded example is a viable, circumferentially oriented preparation from the ventral-lateral region of bovine cervical annulus fibrosus, with the transverse direction in the lamellar plane designated as primary and the second transverse direction recorded separately. Existing work provides a precedent for testing region-specific annular responses under osmotic and time-dependent conditions [31,32]. The new question concerns the later matrix consequence of preserving a measured response, not the existence of those established dependencies.

For this illustrative test, a common longitudinal-strain window is established in preliminary mechanical and viability assessment and fixed before group comparison. All groups are evaluated within that same window; it is not enlarged after a negative result to recover an auxetic finding. The primary exposure descriptor is the qualifying AO span. Recovery is recorded as residual strain 60 min after unloading, together with time to a prespecified return tolerance. Drift is the change from the first stable analytical cycle to the tenth. These are proposed observation windows, not claims of optimal physiological loading.

The biological comparison comprises seven days of repeated, dose-controlled exposure followed by 24 h in a common recovery environment. This timescale is an illustrative starting point: an annulus-directed scaffold study used a seven-day loading period and a subsequent 24 h interval [42], but that precedent does not validate the schedule for native annular explants. Viability, preserved integrity, and adequate endpoint sensitivity must be established within the selected preparation.

The primary structural outcome is persistent matrix compaction, assessed as the change in collagen-occupied volume fraction in a defined tissue region after common-bath equilibration and recovery, relative to baseline or matched controls. Reduced persistent compaction is proposed here as a candidate indicator of matrix preservation, not an established measure of functional adaptability; the latter requires independent functional assessment. Water content and collagen mass are assessed independently so that dehydration-induced concentration is not mistaken for matrix deposition. The hypothesis predicts a smaller persistent increase in this fraction after broader recoverable AO exposure than after varied non-auxetic exposure, with no compensating loss of viability or mechanical integrity. Matrix turnover or cell-state measurements are secondary outcomes that may distinguish passive compaction from cell-mediated remodeling.

An equal or greater increase in compaction in the broad-AO condition would count against the proposed advantage in this system, provided that the comparison can exclude a prespecified meaningful benefit. A nonsignificant result alone is inconclusive when uncertainty remains large. Failure to detect a reproducible auxetic interval would instead count against AO occurrence within the defined preparation and loading window. Preserved AO without a better independent matrix outcome would support mechanical capacity, but not its proposed biological preference.

### 8.6. Future Clinical and Performance Translation

Manual therapy, rehabilitation, movement, and sport provide combinations of tension, compression, muscular force, respiration, displacement, and changing boundaries that could eventually serve as controlled perturbations. Their scientific value would lie in measured tissue mechanics and independent biological or functional outcomes, rather than assigning AO to a named method.

Clinical observations can guide questions about constraints and tissue selection, but visible three-dimensional movement or palpated expansion does not quantify Poisson behavior. Likewise, range of motion reflects ECM, tissue, neural, perceptual, and behavioral contributions [7]. An intervention’s geometry, measured tissue auxeticity, and clinical improvement remain separate observations. Translation becomes persuasive only when a reproducible intervention changes the specified tissue exposure and the predicted independent outcome follows.

## 9. Conclusions

Living connective tissue preserves function by adapting to changing loads rather than maintaining a single shape or mechanical state. Within that physiology, this article proposes that selected tissues may benefit from retaining access to a broad, recoverable auxetic strain interval. Biotensegrity offers an architectural rationale for how such reconfiguration might assist distributed engagement, while established tissue mechanics and ECM biology provide the basis for testing the proposal.

The central claim is reciprocal: matrix state influences the deformation strategies available to a tissue, and repeated use of those strategies may influence its future matrix state. Preserving AO is hypothesized to provide mechanical inputs that sustain adaptability; persistent loss of access could favor restrictive feedback. This is a proposed biological role for an existing class of deformation behavior, not evidence of a newly discovered mechanical law.

The decisive comparison is with a varied non-auxetic exposure of comparable mechanical dose, using an outcome defined independently of AO. The annular example tests reduced persistent compaction as a candidate structural indicator of preservation; functional adaptability requires separate assessment. This distinction allows the hypothesis to be supported, narrowed, or rejected while connecting clinically motivated questions with measurable connective-tissue physiology.

## Figures and Tables

**Figure 3:**
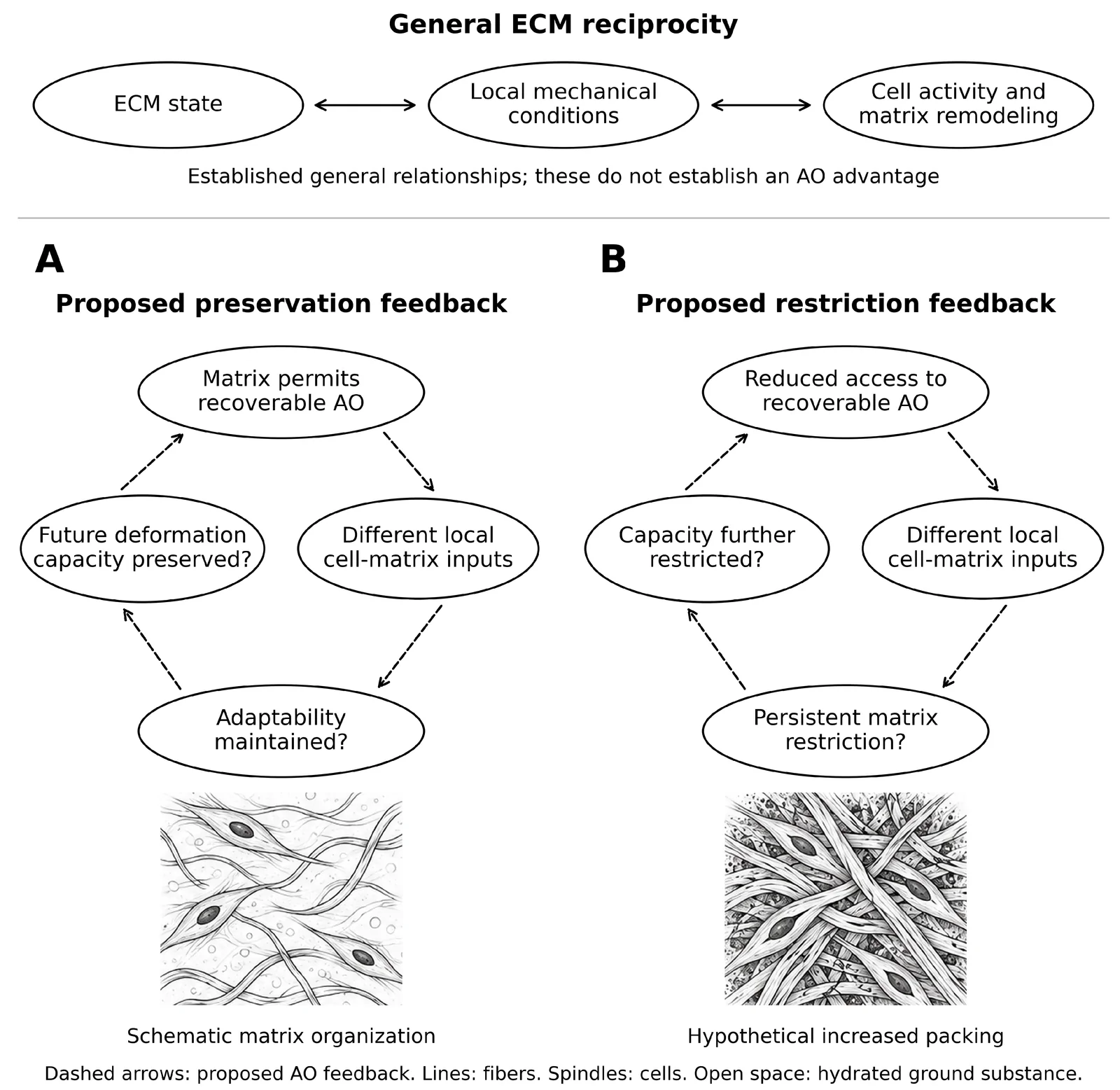
Proposed AO feedback within general ECM reciprocity. The upper pathway summarizes established reciprocal relationships among ECM state, local mechanical conditions, and cell activity/remodeling. Panels (**A**,**B**) propose, respectively, preservation and progressive restriction of AO capacity; all arrows in these lower loops are dashed because the AO-specific relationships remain untested. Matrix drawings indicate fibers, spindle-shaped cells, and hydrated ground-substance space at a common conceptual field size. The denser drawing illustrates possible increased packing, not contamination, diagnosed fibrosis, or a universal pathological appearance. Less persistent post-loading compaction is the independent structural outcome proposed in Section 8.5; preserved AO alone is not evidence of matrix benefit.

**Table 1:** Evidentiary status of claims used in the hypothesis.

Evidence	Domain	What It Supports	What Remains Unestablished
Tendon, annulus, skin and colorectal strain observations [27,28,31,32,33,34]	Biological measurement	Negative-Poisson responses occur in selected preparations and conditions.	Universal tissue auxeticity or a benefit from preserving AO.
Fiber/crimp and annular constitutive studies [29,32]	Modeling informed by architecture or measurements	Candidate mechanisms and interpretations of state-dependent transverse behavior.	Independent proof of the proposed cell–ECM feedback.
Tendon sliding, plantar-fascia thickness/shear-wave changes, and living connective-tissue sliding [30,35,37]	Related tissue observations	Reconfiguration and interaction among tissue components.	A Poisson ratio from observations that do not measure the necessary strains.
Tensegrity geometry, indentation, fracture and pore responses [15,17,18,19,20,21,22]	Engineered structures and materials	Specific auxetic architectures can have useful mechanical properties.	Equivalent benefits in native connective tissue.
AO preservation and restrictive ECM feedback	Proposed biological relationship	A tissue-specific hypothesis with independently testable outcomes.	An established mechanism or explanation for clinical improvement.

**Table 2:** Validation framework for AO occurrence, ECM regulation, and preservation benefit.

Question	Required Comparison	Support	Finding Against or Limiting the Claim
Does AO occur?	Defined tissue, axes, common strain window, separate loading/unloading, repeated cycles.	Resolvable same-sign strain increments, qualifying range and specified recovery.	No reproducible qualifying interval in the stated preparation/window.
Does ECM state regulate AO?	Controlled perturbation, independently verified ECM change, bath/time controls and mechanical covariates.	Reproducible prespecified change in threshold, magnitude, directionality, range, reversibility, hysteresis or recovery.	Covariation alone; no effect with adequate precision; or response explained by uncontrolled co-changes.
Is there a later biological response?	Measured exposure precedes an independently measured cell/matrix outcome.	A reproducible temporal relationship consistent with the proposed pathway.	No predicted response, or a difference attributable to another uncontrolled input. A signaling difference alone is not AO-specific.
Does preservation benefit the matrix?	Broader recoverable AO versus varied non-auxetic and restricted low-variation exposure, with comparable dose.	Less persistent collagen packing after seven-day exposure/24 h recovery, with viability and integrity maintained (Section 8.5): a candidate preservation indicator, not an established measure of functional adaptability.	Equal or worse independent matrix outcome, with precision adequate to test a meaningful advantage.
Do AO measures add useful prediction?	Prespecified baseline including strain exposure, stiffness, pressure and fluid measures; independent validation.	AO descriptors improve prediction of the later outcome beyond that stated baseline.	No reproducible added predictive value. Mediation through fluid/pressure is assessed separately from confounding.
Does the model translate?	Intervention, tissue mechanics, matrix outcome and clinical function measured separately.	Prospective coupling of the specified exposure with an independent biological or functional outcome.	Clinical improvement alone, or AO change without the predicted outcome.

## Data Availability

This theoretical Hypothesis article synthesizes published literature and proposes a conceptual framework for future investigation. No datasets were generated or reanalyzed. The sources supporting the discussion are cited in the article.
